# Incidental para-ureteral aggressive angiomyxoma: a rare case report and literature review

**DOI:** 10.1186/s12894-020-00755-7

**Published:** 2020-11-10

**Authors:** Ming Liu, Ting-Shuai Zhai, Xiao-Feng Zhao, Li-Jin Feng, Xin-Sheng Lyu, Lan-Ting Hu, Zheng-Xin Wang, Wei-Guo Ma, Jian Zhang, Xiao Chen, Bin-Jie Su, Xu-Dong Yao, Jing-Yi Lu, Lin Ye

**Affiliations:** 1 Department of Urology, Karamay Central Hospital, Karamay, 834000 Xinjiang China; 2 Department of Urology, Shanghai Tenth People’s Hospital, Tongji University School of Medicine, No.301, Middle Yan-Chang Rd., Jing-An District, Shanghai, 200072 China; 3 Department of Pathology, Shanghai Tenth People’s Hospital, Tongji University School of Medicine, Shanghai, 200072 China; 4 Department of Radiology, Karamay Central Hospital, Karamay, 834000 Xinjiang China

**Keywords:** Angiomyxoma, Ureter, Case management

## Abstract

**Background:**

Aggressive angiomyxoma (AA) is a rare tumor that typically occurs in the pelvis and perineum, most commonly in women of reproductive age. However, no para-ureteral AA has been reported according to the literature.

Case presentation

We herein describe the first case of para-ureteral AA. A 62-year-old male presented to our institute in March 2017 with a para-ureteral mass that was 15 mm in diameter incidentally. No symptom was observed and laboratory analysis was unremarkable. Magnetic resonance and computed tomography imaging showed a non-enhancing mass abutting the left ureter without causing obstruction. Laparoscopic resection of the mass was performed without injury to the ureter. Pathologic and immunohistochemical results were consistent with AA. Till now, no recurrence was noticed.

**Conclusions:**

We reported a rare case of para-ureteral AA, along with a literature review. Early diagnosis, proper surgical plan and long-term close follow-up is recommended for its high risk of recurrence and malignant potential.

## Background

Aggressive angiomyxoma (AA), synonymously referred to deep angiomyxoma (DAM), is a very rare mesenchymal tumor characterized by local aggressiveness and high risk of recurrence [1]. AA preferentially grows in pelvis and perineum of females aged 30 to 60, with a female-to-male ratio approximately 6:1 [2]. In 1983 Steeper and Rosai first described this disease and named it “aggressive angiomyxoma” [3]. According to the latest WHO classification AA was classified as “tumors of uncertain differentiation”. Pre-operative diagnosis is difficult due to its rarity and lack of typical presenting signs and symptoms. Wide surgical excision is the curative and foremost treatment method for AA [4]. Local recurrence is common even after complete excision. So long-term close follow-up is recommended [5]. However, no para-ureteral AA has been reported. A case of para-ureteral AA of a 62-year-old male is presented herein. To the best of our knowledge, this is the first reported case of para-ureteral AA.

## Case presentation

**Fig. 1:**
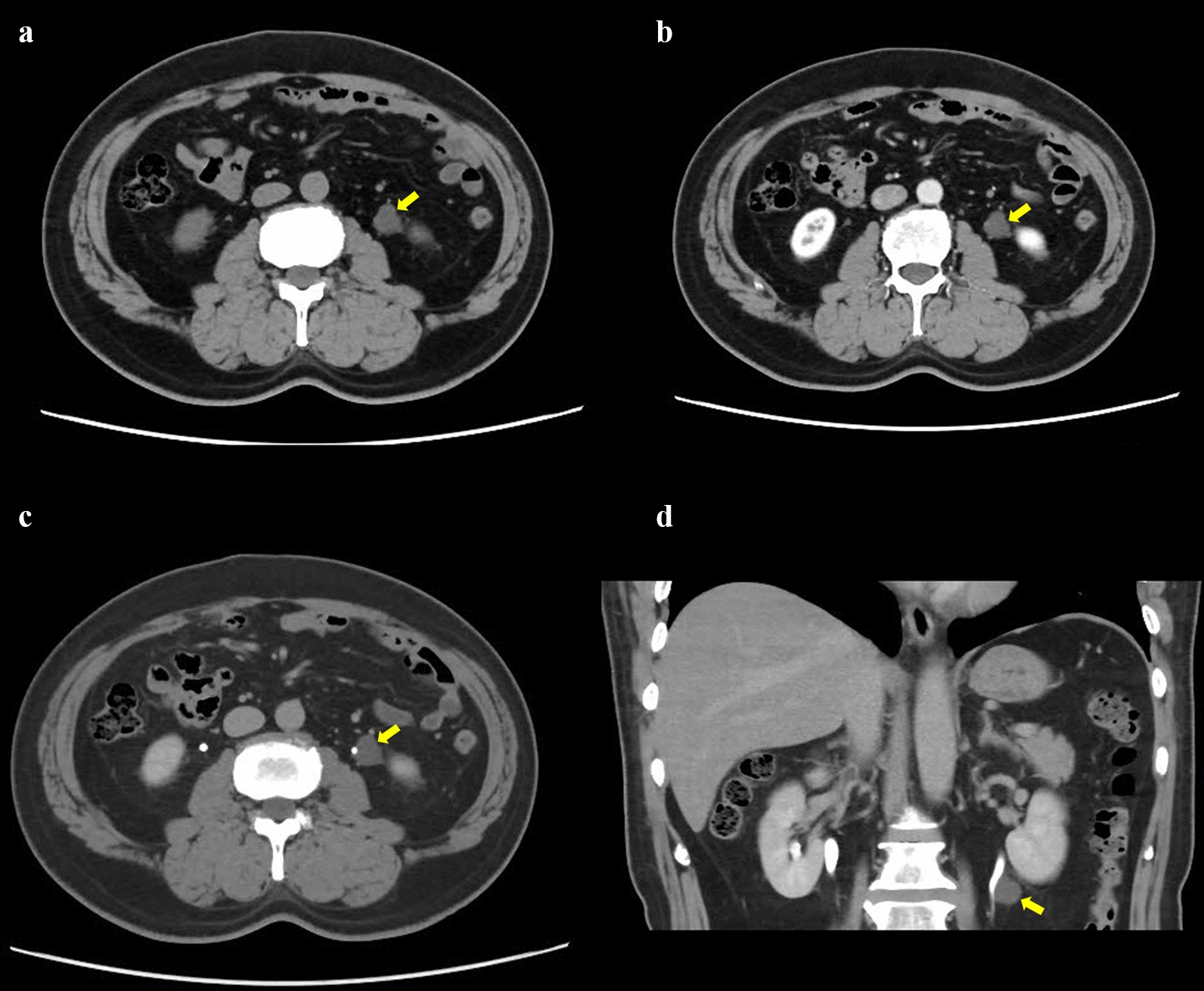
Axial (**a**–**c**) and coronal (**d**) contrast-enhanced CT of the abdomen. CT imaging showed the mass abutting the left ureter was 15 mm in diameter without causing obstruction (**a**, **b**), which could been obviously seen in the excretory phase (**c**). The mass was located between the left ureter and the lower pole of the left kidney (**d**)

**Fig. 2:**
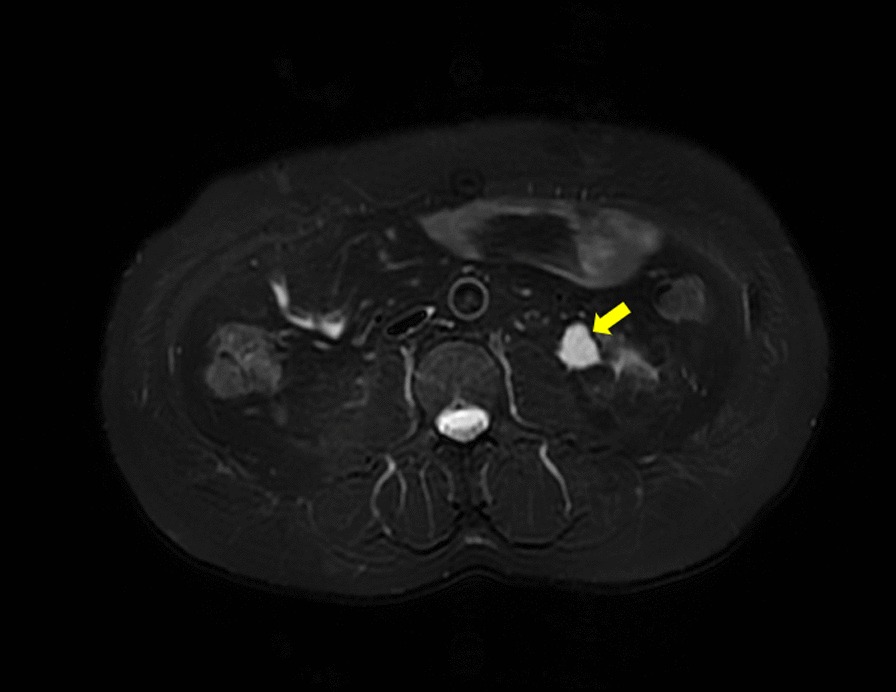
MRI appearances of the tumor demonstrated hyperintensity on T2-weighted images

A 62-year-old male was hospitalized in March 2017 with a para-ureteral mass that was 15 mm in diameter found through a routine check-up incidentally. There was no symptom. On physical examination no abnormality was detected. Laboratory tests (blood routine test, urine routine test, biochemical tests) including tumor markers (β-HCG, CA125, CA242, CA15-3, CA199, CEA, AFP, NSE, CYK-19, SCC, t-PSA, f-PSA) were unremarkable. CT imaging showed an irregular-shaped non-enhancing mass abutting the left ureter was 15 mm in diameter, with unclear boundaries to the ureter, hypodense relative to muscle (Fig. 1). MRI appearances of the tumor demonstrated hyperintensity on T2-weighted images (Fig. 2). And No hydronephrosis was observed in the imaging.

**Fig. 3:**
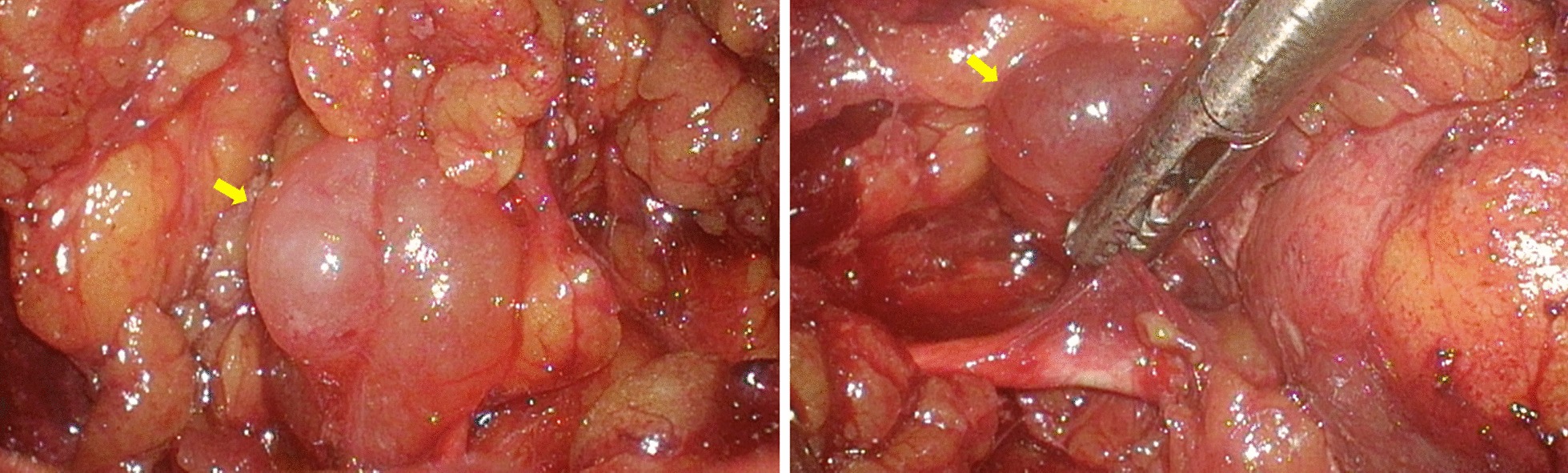
Surgical photograph. A well-defined and gelatinous cystic mass, 2.3 × 2.0 × 1.0 cm in size, with a smooth surface was observed abutting the upper left ureter with a stent inside

**Fig. 4:**
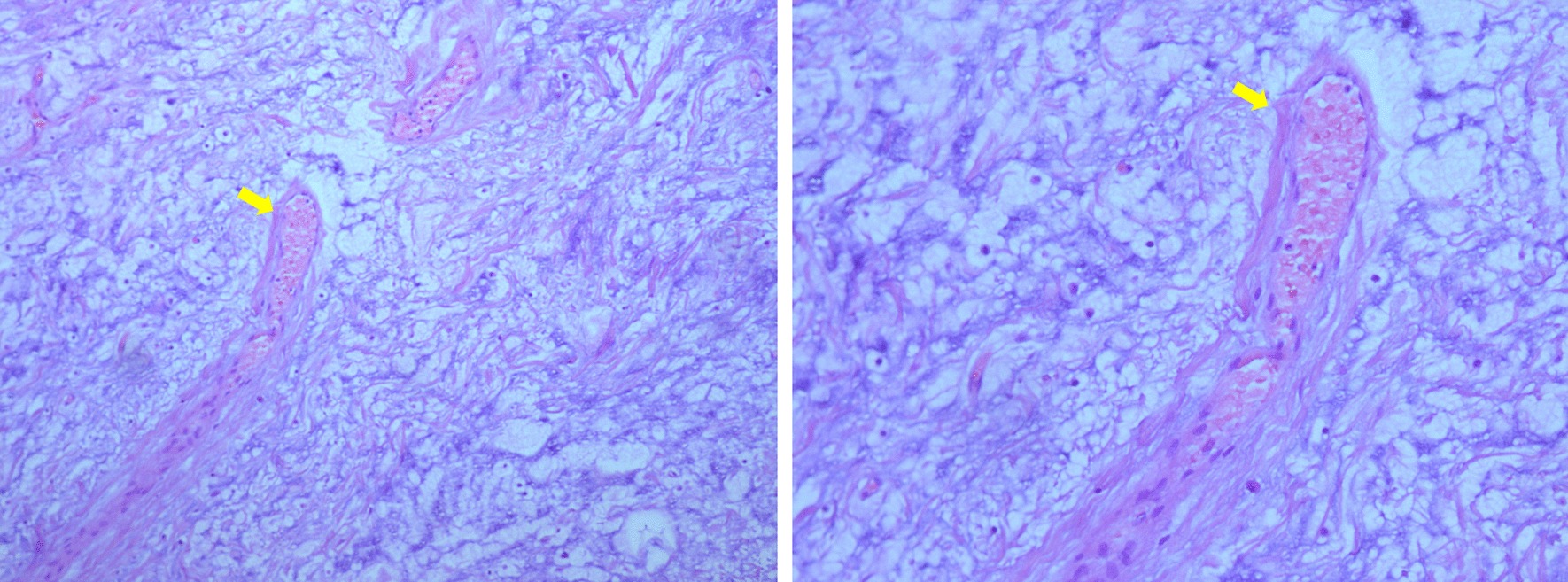
Pathological photos. Histopathological analysis revealed spindle-shaped cells in loose myxoid stroma with variable calibre blood vessels

Laparoscopic resection of the tumor was performed. The intraoperative situs revealed a well-defined and gelatinous cystic mass with a smooth surface abutting the upper left ureter, 2.3 × 2.0 × 1.0 cm in size (Fig. 3). Histopathological analysis revealed spindle-shaped cells in loose myxoid stroma with variable calibre blood vessels (Fig. 4), consistent with AA. Mitotic figures were absent. Immunohistochemically the tumor cells were positive for Vimetin, Ki-67(weakly, 0–2%) and S-100(focally), while negative for Desmin, SMA, CD34, EMA and CK(AE1/AE3). The postoperative period was uneventful. After 30-month follow-up, no evidence of recurrence was noted. The patient has been informed to keep the follow-up because of the high risk of recurrence.

## Discussion and conclusions

Aggressive Angiomyxoma, as a rare soft-tissue neoplasm, seldom occurs in urinary system especially in ureter. We reviewed the latest 100 AA patients published, analyzed the localization and found no para-ureteral AA (Additional file 1: Fig. 1). “Aggressive” refers to the nature of its local infiltration and recurrence [6]. Because of its rarity, the preoperative misdiagnosis rate of AA is rather high and the pathogenesis is poorly understood. Both of proper management and prognosis have not been investigated very well. In the present case, the patient benefited from early detection, proper treatment and regular follow-up. The literature review focused on the clinical characteristics, pathological feature and the treating effect.

Most patients have no obvious discomfort. The mass often grows at the perineal or pelvic region [1]. If the tumor is large enough, compressive symptoms may occur. Imageological examinations of AA have low specificity. Color doppler ultrasound usually indicates a hypoechoic or cystic mass [7]. CT often shows a well-defined mass, hypodense to muscle [9]. MRI revealed T1 hypointensity and T2 hyperintensity and a characteristic “swirled” appearance inside the mass can be observed [8]. Furthermore, MRI can identify the infiltrating borders and relationship between the tumor and surrounding organs, which could help surgeons make optimal surgical plan. Pathological examination is the gold standard for the diagnosis of AA. On gross examination, the soft tumors are white and have a rubbery consistency with a gelantinous, translucent and glistening cut surface [7]. Microscopically, stellate to spindle-shaped tumor cells with ill-defined cytoplasmic borders, fibromyxoid stroma and hyalinized thin-to-thick wall vessels can always observed. Mitotic figures are always absent [8, 9]. Immunohistochemically the tumor has no specific marker, showing diffuse positivity for Vimentin, SMA, Desmin, CD-34, estrogen receptor and progesterone receptors. While, S-100, cytokeratins and CD68 are usually negative [1, 7].

At present surgical resection of the tumor with wide free margin is the most effective treatment [8]. However, surgical plan differs in different patients. For the small and superficial well-defined tumors, wide local excision is suitable. For the large and deep tumors but with clear borders to the adjacent tissue, efforts should be made to perform complete excision. While, for those extensively and deeply invading the surrounding vital organs, if complete excision is infeasible or severe surgical injuries may happen, incomplete resection combined with adjuvant therapy are recommended [10]. Zugail et al. reported a 35-mm AA mimicking an upper tract urothelial carcinoma (UTUC) of the right pelvic ureter treated with nephroureterectomy [11]. In our case, the tumor was small and well-defined, so wide local excision was performed successfully. If the tumor grew larger and invaded the ureter, it would not be easy to perform complete excision. Hormonal treatment, including raloxifene, tamoxifen, and gonadotropin-releasing hormone analogs, is sometimes used to shrink the tumor before surgery. Moreover, hormonal treatment is necessary especially for the residual or recurrent tumors [10]. However, chemotherapy, radiation and embolization are generally considered to be of limited use due to the tumor's low mitotic activity and numerous feeding vessels [8].

Because AA has a high recurrence rate (36–72%) [2, 12], postoperative surveillance is necessary. MRI is considered as a useful tool to detect recurrence. The factors affecting risk of recurrence remain unclear. Some researchers hold that incomplete surgical resection increases the risk of recurrence [2] but remains unproven. According to the literature, most of patients have had a relatively favorable prognosis, despite local multiple recurrences and infiltration to adjacent organs and tissues. However, two metastasizing cases were reported, ending in death [13, 14], which highlight the need to consider it as potentially malignant in a small percentage of cases.

From our perspective, diagnosis mainly depends on CTU and MRI. Complete resection of tumor should be performed and partial pelvis or ureter should also be resected if necessary. During follow-up, US can be used to detect hydronephrosis and MRI can be used to detect recurrence. To conclude, we reported a rare case of para-ureteral AA, along with a literature review. CT or MRI is helpful to detect early stage para-ureteral AA, which can be removed intactly and safely by laparoscopy. Thus, it can help increase the cure rate and decrease the recurrence rate and mortality. Early diagnosis, proper surgical plan and long-term close follow-up is recommended for its high risk of recurrence and malignant potential.

## Supplementary information

**Additional file 1: Fig. 1**. Location distribution of the latest 100 aggressive angiomyxomas.

## Data Availability

The datasets used or analyzed during the current study are available from the corresponding author on reasonable request.

## Abbreviations

AA: Aggressive angiomyxoma
DAM: Deep angiomyxoma
CT: Computerized tomography
MRI: Magnetic resonance imaging
