# Supplementary figures and images for: Incidental para-ureteral aggressive angiomyxoma: a rare case report and literature review
Source: BMC Urol. 2020 Nov 10;20:182. doi: 10.1186/s12894-020-00755-7 (PMC7653999; doi:10.1186/s12894-020-00755-7)

Supplementary figure 1 Location of the latest 100 aggressive angiomyxoma patients published.


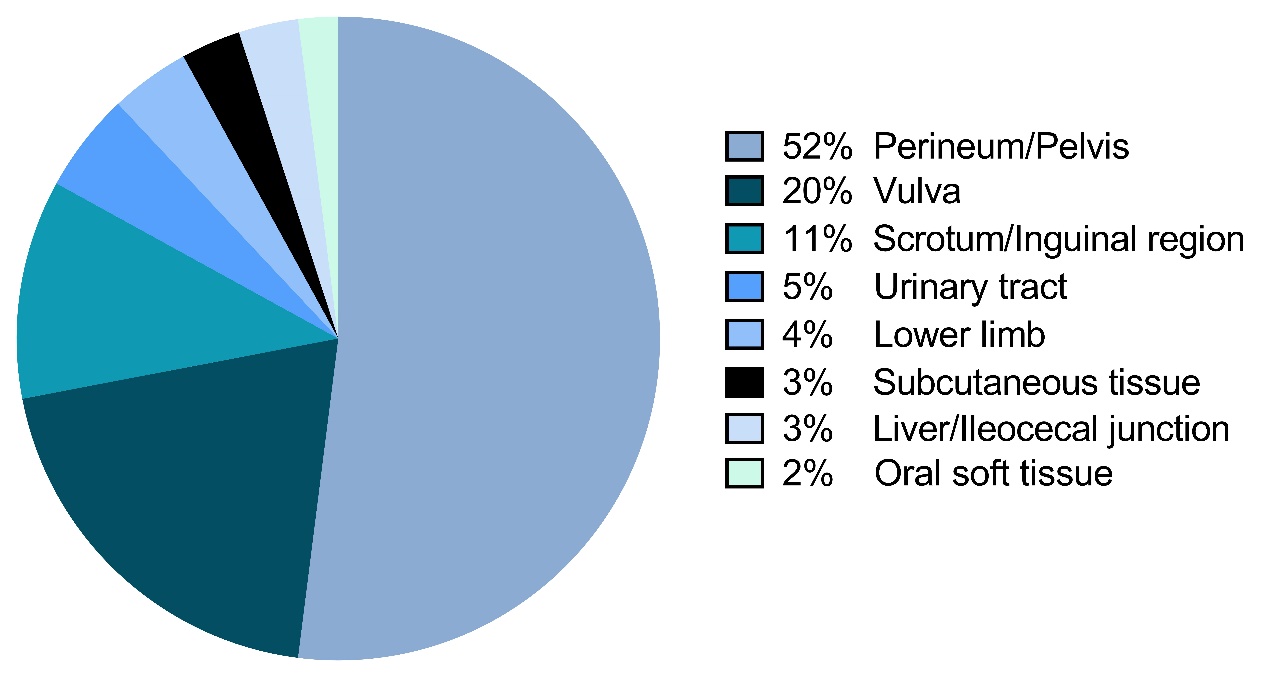

Supplement: Supplementary file 1 — Additional file 1: Fig. 1. Location distribution of the latest 100 aggressive angiomyxomas. [file 12894_2020_755_MOESM1_ESM.docx]
